# *Vaccinium* as Potential Therapy for Diabetes and Microvascular Complications

**DOI:** 10.3390/nu15092031

**Published:** 2023-04-23

**Authors:** Hui Huang, Yayong Luo, Qian Wang, Yihan Zhang, Zhongxia Li, Ruikun He, Xiangmei Chen, Zheyi Dong

**Affiliations:** 1 National Clinical Research Center for Kidney Diseases, State Key Laboratory of Kidney Diseases, Beijing Key Laboratory of Kidney Disease Research, First Medical Center of Chinese PLA General Hospital, Nephrology Institute of the Chinese People’s Liberation Army, Beijing 100853, China; 2 School of Clinical Medicine, Guangdong Pharmaceutical University, Guangzhou 510006, China; 3 BYHEALTH Institute of Nutrition & Health, No. 3 Kehui 3rd Street, No. 99 Kexue Avenue Central, Huangpu District, Guangzhou 510663, China

**Keywords:** diabetes mellitus, diabetic kidney disease, diabetic retinopathy, Ericaceae, *Vaccinium*

## Abstract

Diabetes mellitus is one of the most critical global health concerns, with a fast-growing prevalence. The incidence of diabetic vascular complications is also rapidly increasing, exacerbating the burden on individuals with diabetes and the consumption of public medical resources. Despite the overall improvements in the prevention, diagnosis, and treatment of diabetic microvascular complications in recent years, safe and effective alternative or adjunctive therapies are urgently needed. The mechanisms underlying diabetic vascular complications are complex, with hyperglycemia-induced oxidative stress and inflammation being the leading causes. Therefore, glycemic control, antioxidation, and anti-inflammation are considered the main targets for the treatment of diabetes and its vascular comorbidities. *Vaccinium* L. (Ericaceae) is a genus of plants enriched with polyphenolic compounds in their leaves and fruits. *Vaccinium* and its extracts have demonstrated good bioactivity in reducing blood glucose, oxidative stress, and inflammation, making them excellent candidates for the management of diabetes and diabetic vascular complications. Here, we review recent preclinical and clinical studies on the potential effect of *Vaccinium* on ameliorating diabetes and diabetic complications, particularly diabetic kidney disease and diabetic retinopathy.

## 1. Introduction

Due to rapid urbanization and population aging, people are changing their dietary and nutritional habits, leading to an increase in the global prevalence of diabetes mellitus (DM) [1,2]. Diabetes is one of the world’s greatest public health challenges [3], particularly in developing countries [4]. Type 2 diabetes accounts for approximately 90–95% of all diabetes cases and is strongly associated with an unhealthy lifestyle, such as energy-rich diets and sedentary behaviors [5]. Patients with diabetes are prone to microangiopathic complications and have a higher risk of mortality [6]. Microvascular complications of diabetes continue to compromise the quality of life of patients with diabetes. The most representative of these are diabetic kidney disease (DKD) and diabetic retinopathy (DR). However, the inclusion of diabetic neuropathy as a microvascular complication has recently been challenged. In China, an estimated 24.3 million people with diabetes have chronic kidney disease (CKD) [7]. In the United States, the number of people with diabetes who have started treatment for end-stage renal disease (ESRD) has increased considerably from more than 40,000 in 2000 to more than 50,000 in 2014 [8]. Mortality is approximately 30 times higher in patients with diabetic nephropathy compared to in those without [9]. DR is the leading cause of low vision and blindness in patients with diabetes, with an annual incidence of up to 12.7% [10]. These microvascular lesions also increase the risk of all-cause and cardiovascular disease (CVD) mortality [11,12]. Owing to the increasing incidence of diabetes and the associated socioeconomic impact of diabetes-related complications, there is an urgent need to improve the management of patients with diabetes.

Inflammation and oxidative stress play key roles in the pathogenesis of diabetes and its microvascular complications. Metabolic abnormalities in diabetes lead to the excessive production of mitochondrial superoxide in the endothelium of small blood vessels. This increase in superoxide production activates five major pathways: overactivity of the hexosamine pathway, polyol pathway flux, activation of protein kinase C isoforms, increased formation of advanced glycation end products (AGEs), and increased expression of the AGE receptor and its activating ligands [13]. This review focuses on the management of two microvascular complications, DKD and DR. Although angiotensin-converting enzyme inhibitors (ACEI)/angiotensin receptor blockers (ARB) and sodium–glucose cotransporter 2 (SGLT2) have been widely used for DKD treatment, as well as laser and injection therapy for DR, safer and more acceptable adjuvant treatments are urgently needed.

Natural compounds in plants provide a safer and more effective way to treat diabetes and its microvascular complications than drugs [14]. The *Vaccinium* L. (Ericaceae) genus, which includes a range of shrubs and dwarf shrubs, contains a wide diversity of economically and culturally important berry crop species [15,16]. These fruits contain various volatile organic compounds [17]. *Vaccinium* reportedly contains phenolic compounds, pectin, vitamins, and sugars [18,19]. Anthocyanins (ANT) are found in the genus *Vaccinium* L. (Ericaceae) [20]. ANT is metabolized in the body as follows: it is absorbed in the gut and then enters the liver through the portal vein, where it is metabolized, secreted, and reabsorbed [21].

*Vaccinium*, including its fruits, flowers, and leaves, is widely used in traditional medicine, especially for the empirical treatment of diabetes, vision-related ailments, and several cardiovascular disorders [19,22,23]. Phytoconstituents of *Vaccinium* have antioxidant, anti-inflammatory, antibacterial, anti-obesity, anti-cancer, anti-diabetic, eye-cardioprotective, and neuroprotective activities [18,24,25,26,27,28,29]. The *Vaccinium* extract provides an avenue for the development of new drugs. 

In this review, we aimed to describe the current knowledge of the anti-diabetic and anti-microvascular complications (DKD and DR) properties of some *Vaccinium* species (focusing on four popular species of *Vaccinium*, referring to the fruits, if not specified), including the mechanisms of action, experimental study results, and some positive clinical evidence.

## 2. Materials and Methods

The Web of Science Core Collection (WoSCC), Scopus, and PubMed databases were used for literature searches. The search ended on 1 February 2023. Combinations of several search terms such as “*Vaccinium*” “bilberry,” “anthocyanins,” “flavonoids,” “polyphenols,” “diabetes,” “diabetic kidney disease,” “diabetic retinopathy,” “in vitro,” “in vivo,” “clinical trial,” “antioxidant,” “anti-inflammatory,” and “immune-modulatory” were used. Bibliometric and visual analyses were performed using a scholarly search on the website.

## 3. Research Status of *Vaccinium* in Treating Diabetes Mellitus and Its Microvascular Complications

**Figure 1:**
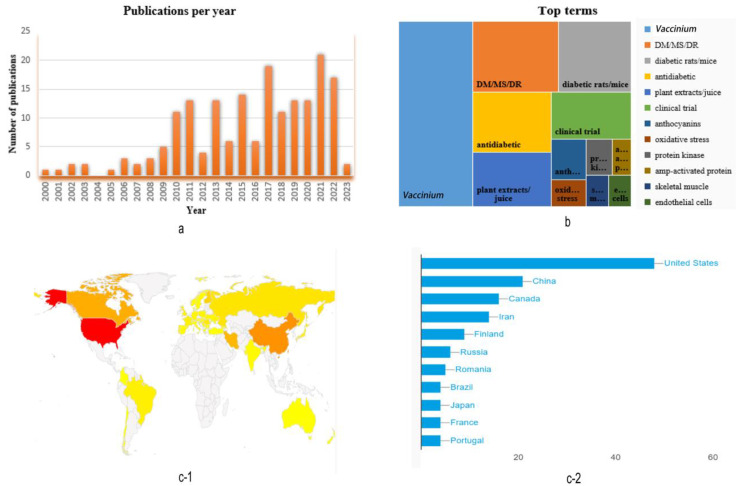
Research status of *Vaccinium* in treating diabetes mellitus and its microvascular complications. (**a**) Publications per year; (**b**) Top terms; (**c-1**,**c-2**) Regional distribution.

The number of publications focusing on *Vaccinium*, diabetes, and its complications has rapidly increased over the last 10 years (Figure 1a). High-frequency terms focused on *Vaccinium*, DM, metabolic syndrome (MS) or DR, plant extracts or juice, anthocyanins, rat and mouse animal experiments, clinical trials, anti-diabetic, and oxidative stress (Figure 1b). In terms of regional distribution, the Americas, Asia, and Europe, represented by the United States, Canada, China, Iran, Russia, and Finland, respectively, have made significant contributions to the research in this field (Figure 1(c-1,c-2)).

## 4. Vaccinium

### 4.1. General 

The *Vaccinium* L. (Ericaceae) genus, consisting of approximately 450 species, contains a range of terrestrial or epiphytic shrubs and dwarf shrubs that mainly grow in cooler areas across Europe, Southeast and Central Africa, North and Central America, and Asia [15,30]. Most *Vaccinium* fruits are edible, and some have a long history of human consumption. *V. corymbosum* (blueberry), *V. oxycoccos* (cranberry), *V. macrocarpon* (American cranberry), *V. myrtillus* (bilberry), *V. Arctostaphylos* (bearberry), and *V. vitis idaea* (lingonberry) are the species of *Vaccinium* most investigated [17]. Arevka illustrated the differences between four common species: bilberry, blueberry, lingonberry, and cranberry [15].

Leaves and fruits have been widely used in traditional medicine for the treatment of stomatitis; diabetes; renal stones; and intestinal, liver, and urinary tract disorders, as early as the 18th century [31]. Some *Vaccinium* species were domesticated in the 20th century and are now cultured on a large scale in several regions worldwide as economic fruits. 

### 4.2. Chemical Profile

The chemical components of *Vaccinium* have been extensively investigated in several studies. Polyphenolic components, including ANT (cyanidin, malvidin, and delphinidin), flavonoids (quercetin, isoquercetin, kaempferol, apigenin, and myricetin), phenolic acids (gallic, *p*-coumaric, cinnamic, syringic, ferulic, and caffeic acids), and ellagitannins, are considered the main bioactive compounds of *Vaccinium* [19].

ANT is the primary phytochemical characteristic of *Vaccinium*. To date, more than 35 anthocyanin glycosides have been identified in *Vaccinium*, with the principal anthocyanins varying among species (Table 1). In addition, more than 50 other flavonoids, mainly flavanols and pro-anthocyanidins, have been identified in *Vaccinium*, and their profiles vary between species [30]. 

### 4.3. Bioactivity

Phytochemicals from several *Vaccinium* species exhibit good activity in multiple biofunctions. The enrichment of polyphenolic compounds leads to a strong antioxidant effect, which is the most acknowledged bioactivity of these berries [20]. Similarly, high concentrations of ANT and flavonoids contribute to the anti-inflammatory effects of *Vaccinium*. As many types of tissue damage are closely associated with oxidative stress and inflammation, *Vaccinium* demonstrates therapeutic potential under multiple pathological conditions, such as diabetes and diabetic vascular damage [36,37].

Moreover, *Vaccinium* has antimicrobial, anticarcinogenic, cardiovascular protective, vision improvement, and anti-neurodegenerative effects, which have been described in detail elsewhere [23,31,38,39,40]. Berries have the potential to reduce metabolic and cardiovascular risk [40,41]. Similarly, the intake of blueberries has been associated with a reduced risk of cardiovascular disease, death, and type 2 diabetes (T2D), as well as improved weight maintenance and neuroprotection in some epidemiological studies [42,43]. In addition, cranberries have special effect against urinary tract inflammation, tooth decay, periodontitis, and *Helicobacter pylori* infection of the stomach [44].

## 5. Effects of Oxidative Stress and Inflammation on Diabetes and Its Microvascular Complications

### 5.1. Abnormalities of Glucose and Lipid Metabolism

Type 2 diabetes mellitus (T2DM) accounts for more than 90% of diabetes cases and is typically characterized by abnormally high blood glucose levels and insulin resistance [5]. The maintenance of hyperglycemia leads to the production of mitochondrial superoxide in endothelial cells of small vessels. As a lack of insulin stimulates malonyl coenzyme A (CoA) production, the insulin receptor (IR) increases the oxidation of free fatty acids (FFAs) in endothelial cells, leading to increased superoxide production by the mitochondrial electron transport chain [13]. Mitochondrial superoxide production activates five pathways involved in diabetic microvascular pathogenesis [13]. During the development of diabetic complications, hyperglycemia acts synergistically with other risk factors (obesity, hypertension, and dyslipidemia) to accelerate the presentation of histopathological features of diabetes [45]. 

In contrast, cellular oxidative stress and disequilibrium of REDOX homeostasis are common features in patients with DM [46,47,48]. The bioavailability of nitric oxide (NO) and uncoupling endothelial nitric oxide synthase (eNOS) are two major factors that lead to changes in vascular reactivity and the production of reactive oxygen species (ROS) and reactive nitrogen species (RNS) [49,50,51]. In addition, the main factor in oxidative stress is the imbalance between promoting enzymes (e.g., NADPH oxidase complex (Nox), cytochrome 450, xanthine oxidase, and myeloperoxidase) and antioxidant enzymes (e.g., superoxide dismutase, catalase, and glutathione (GSH) peroxidase) [52,53,54]. Kinases and transcription factors involved in many inflammatory and oxidative stress responses activate intracellular signaling pathways that lead to the production of pro-oxidative, pro-vascular and pro-inflammatory factors, such as chemokines, cytokines, pro-oxidant enzymes, extracellular matrix proteins, growth factors, and adhesion molecules [55,56,57].

In conclusion, oxidative stress and inflammation induced by abnormal glucose and lipid metabolism are the major pathogenic factors associated with diabetic complications. 

### 5.2. Diabetic Kidney Disease

DKD is characterized by thickening of the glomerular basement membrane (GBM), dilation of the mesangial matrix, the formation of characteristic Kimmelstiel–Wilson nodules, and a progressive decline in albuminuria and glomerular filtration rate (GFR) [58]. It is characterized by metabolic disturbances and hemodynamic abnormalities caused by hyperglycemia. Some of these pathways involve the formation of AGEs, renin-angiotensin-aldosterone system (RAAS), aldol reductase activation, polyol pathway activation, protein kinase C (PKC), ROS, an increase in some cytokines, connective tissue growth factor (CTGF), and the activation of transforming growth factor beta 1 (TGF-β1) [59,60,61]. Elevated ROS levels due to hyperglycemia are central to the pathogenesis of DKD. In diabetes, the main sources of ROS are NOX, AGE, and polyol chains [62]. Oxidative stress can directly damage the podocytes, mesangial and endothelial cells, leading to albuminuria and tubulointerstitial fibrosis [63,64]. Innate immunity is involved in the occurrence and development of DKD. Mechanistically, TLR4 is overexpressed in DKD and is negatively correlated with renal function and positively correlated with HbA1c levels [65,66]. In addition, TLR2 [65,67] and NLRP3 inflammasome activation of interleukin-1β (IL-1β) [65,68,69] play a major role in metabolic stress in DKD. The pathogenesis of diabetic nephropathy is influenced by a combination of multiple factors, and there is a large amount of overlap between pathways and intermediaries. For example, oxidative stress can indirectly activate other pathways to cause damage, while other pathogenic pathways can cause damage through oxidative stress. Therefore, the exact pathogenic and molecular mechanisms of DKD remain unclear.

At present, strict control of blood pressure and blood glucose and inhibition of the RAAS by ACEI or ARB are the main methods for the treatment of DKD. The introduction of new glucose-lowering agents, finerenone, and sodium-dependent glucose transporter 2 (SGLT-2) has dramatically changed the treatment landscape of T2D [70,71]. The clinical advantages associated with the use of SGLT2 inhibitors include antifibrotic effects due to the correction of oxidative stress and inflammation, autophagy, and modulation of mitochondrial function [72]. The EMPA-REG outcome study (Empagliflozin Cardiovascular Outcome Event Trial in Type 2 Diabetes Mellitus Patients) showed that empagliflozin reduces cardiovascular death or worsens kidney disease by 39% [73]. The CANVAS study (Canagliflozin Cardiovascular Assessment Study) has also shown that canagliflozin reduces cardiovascular and renal outcomes [74].

However, these methods only delay the progression of DKD and do not prevent or reverse its progression to ESRD [61]. Therefore, new drugs targeting the pathological mechanisms of DKD, such as oxidative stress and inflammation, have become the main focus of new therapies to treat DKD [75].

### 5.3. Diabetic Retinopathy

DR is classically divided into two major categories: non-proliferative (NPDR) and proliferative (PDR). NPDR is characterized by defects in the retinal vasculature, including hemorrhage, such as punctate bleeding and spot bleeding [76,77,78], hard exudation [77,79], flocculent spot [76,77,79,80], microaneurysms [76,77,78,79], and vascular leakage [79,80]. The main feature of PDR is pathological retinal angiogenesis [81], which includes the growth of new abnormal blood vessels from the existing vascular network and the generation of retinal neovascularization [77,82,83]. The progression of NPDR to PDR is characterized by increased expression of ischemia, hypoxia, and pro-angiogenesis. Angiogenic growth factors include vascular endothelial growth factor (VEGF), fibroblast growth factor-2 (FGF-2), platelet-derived growth factor (PDGF), and angiopoietin-2 (Ang-2), which can activate abnormal retinal vessels, causing them to protrude into the preretinal space [81,82,83,84,85]. Inflammation has been described as a mechanism of DR [86,87]. The key cytokines in this process are IL-1β, IL-1-dependent IL-6, IL-8, and TNF-α, as well as monocyte adhesion to the endothelial wall and then chemotaxis to the subendothelial space [65,86,88]. In end-stage diabetic retinopathy, severe hypoxia leads to neovascularization, vitreous hemorrhage, and retinal detachment [89].

Although the mechanism behind this has not been fully elucidated, oxidative stress has been shown to be a key factor in this process [90]. In the ischemic state, oxidative stress, GSH, lipid peroxide, malondialdehyde and superoxide dismutase levels increase, while antioxidant levels decrease, thereby inducing oxidative damage to the retina [91]. According to in vitro experiments, elevated superoxide levels were observed under hyperglycemic conditions and increased hydrogen peroxide content was observed in retinal cells [92,93]. Oxidative stress can damage cell membranes and induce apoptosis, microvascular damage, and barrier damage, ultimately leading to the development of DR.

Intraocular treatments for DR include laser photocoagulation, intravitreal injections of anti-vascular endothelial growth factor (VEGF) agents and steroids, and vitreoretinal surgery [77,94]. Although these treatments may slow the progression of DR blindness, they are not effective in treating the disease and have considerable side effects [95,96]. Therefore, there is an urgent need to identify alternative or adjuvant treatments to prevent DR and slow its progression.

## 6. Experimental Study on Diabetes Mellitus and Diabetic Microvascular Complications Treatment with *Vaccinium* Extract

### 6.1. Regulation of Glucose and Lipid Metabolism Disorders

Phenolics target the key pathways involved in carbohydrate metabolism and hepatic glucose homeostasis, including glycogenesis, glycolysis, and gluconeogenesis [30]. The mechanism of the hypoglycemic action of *Vaccinium* may be mediated in part by interference with enzyme action, and polyphenols in *Vaccinium*, such as flavonoids and tannins, can inhibit α-amylase and α-glucosidase [97,98]. Intestinal α-glucosidase breaks down oligosaccharides and disaccharides into monosaccharides suitable for absorption, and *Vaccinium* slows the release of glucose into the bloodstream [99]. A study in prediabetic and diabetic mice showed that bilberry extracts inhibited the activities of α-glucosidase and α-amylase and prevented postprandial hyperglycemia by slowing the rate of carbohydrate digestion [100]. Several polyphenols, such as quercetin, resveratrol, and epigallocatechin-3-gallate, are transported to the plasma membrane mainly through the activation of the protein kinase (AMPK) pathway, thereby enhancing glucose uptake in muscle and adipocytes [98]. 

There is scientific evidence that the intake of polyphenol-rich fruits, which improve diet-induced insulin resistance, is beneficial for the health of obese animals [101]. Mice treated with bilberry extract (BBE) show a considerable reduction in blood glucose levels [102]. One study showed that rats treated with bilberry extract (nonacylated anthocyanins extract from bilberries: NAAB) for 8 weeks had decreased fasting plasma glucose levels [103]. Male mice treated with lingonberry for 8 weeks showed reduced fasting and postprandial hyperinsulinism, improved insulin sensitivity, and enhanced hepatic insulin clearance. In a diet-induced obesity (DIO) mouse model, bilberry treatment at 125, 250, and 500 mg/kg/day significantly reduced blood glucose levels by 28%, 25%, and 17%, respectively. In this model, lingonberry considerably reduced blood glucose and insulin levels [104]. In another study, mice fed lingonberry showed improvements in blood sugar and liver function, along with a reduction in inflammation [105].

*Vaccinium* is beneficial in reducing adipose tissue inflammation in models of metabolic syndrome [106,107,108,109]. In addition, dried bilberry (*Vaccinium myrtillus* L.) slowly reduces serum cholesterol and delays the adverse consequences of high-fat diet-induced lipid and glucose metabolism caused by high-fat diet [110]. Lingonberry (*Vaccinium vitis-idaea* L.) treatment of hypertrophic adipocytes resulted in reduced lipid accumulation and triglyceride (TG) content, and downregulated expression of lipogenic genes for fatty acid and TC synthesis, such as fatty acid synthase (FAS), adipocyte protein 2 (aP2), and diacylglycerol acyltransferase-1 (DGAT1) [111]. Bilberries, lingonberries, cranberries, and blueberries can regulate glucose and lipid metabolism [112,113,114,115,116,117,118,119,120,121]. Experimental studies on diabetes mellitus treatment with *Vaccinium* extracts are listed in Table 2.

In summary, evidence from preclinical studies suggests that the *Vaccinium* extract is beneficial for controlling blood glucose, lipids, insulin resistance, oxidative stress, and inflammation.

### 6.2. Treating DR and DKD 

The results of experimental studies on the treatment of DR and DKD with *Vaccinium* extracts are listed in Table 3. An increasing number of consumers and scientists are realizing the visual benefits of ANT-rich Vaccinium, and ANT is currently used in ophthalmology to prevent diabetic retinopathy and improve vision [137]. Blueberries contain abundant ANT, which is beneficial for eye health. Blueberry extracts from northeast China, in which Cy-3-glu is the most abundant, ameliorated oxidative stress-induced blood retinal barrier (BRB) damage in the retina [138]. The total anthocyanin content at the optimal dose was estimated to be 36 mg/kg [138]. In another study, blueberry anthocyanin extract (BAE) prevented the progression of DR via molecular regulation of ROS/endoplasmic reticulum stress (ERS) and the miR-182/8-oxoguanine-DNA glycosylase (OGG1) axis [139]. One study showed that blueberry ANT can protect retinal cells from diabetes-induced inflammation and oxidative stress through the regulation of Nrf2/HO-1 signaling [140]. ANT in blueberries protects human retinal capillary endothelial cells through anti-inflammatory and anti-oxidative mechanisms because malvidin-3-glucoside can reduce angiogenesis in a DR-induced cell model by inhibiting the Akt pathway and reducing VEGF levels, inhibits the protein kinase B pathway and decreases the level of VEGF to reduce angiogenesis in the DR-induced cell mode [141]. Blueberries and bilberries can be used to develop nutritional supplements for the prevention of diabetic retinopathy. Bilberry anthocyanosides promote the synthesis and regeneration of rhodopsin, increase the sensitivity of the retina to changes in light intensity, improve the blood supply to the retina, visual acuity and dark adaptation [142]. Bilberry extract treatment also decreased the expression of DR markers, such as degradation of zonula occludens-1, occludin, claudin-5, and retinal VEGF, and prevented or delayed the onset of early diabetic retinopathy in diabetic rats [143].

There are few experimental studies of Vaccinium in the treatment of DKD. DIAVIT, a natural sea buckthorn and Vaccinium myrtillus extract, manipulates gene splicing and expression to treat type II mouse model of diabetic nephropathy in mice. DIAVIT, particularly delphinidin, changes vascular endothelial growth factor A (VEGF-A) splicing and rescues the diabetic nephropathy (DN) phenotype [144]. One study showed that key indicators of renal failure, such as urine color, turbidity, and total protein, were considerably reduced in cats with chronic kidney disease receiving a nutritious diet containing 0.0371% cranberries [145]. The chemical components of Vaccinium include ANT, flavonoids, ellagitannins, and phenolic acids. ANT are polyphenolic compounds present in various foods and play an important role in treating DKD. A study showed that prevention of the progression of DKD by ANT could be related to the regulation of amino acid metabolism. After treatment with ANT, fasting blood glucose levels, glomerular fibrosis scores, glomerular lesion perimeters, and kidney function (urine creatinine and Cystatin C) were considerably alleviated in DKD mice [146]. Another study found that body weight, systolic blood pressure, C-peptide, serum insulin, glycosylated hemoglobin A1c, and elevated fasting blood glucose levels in diabetic mice were remarkably reduced by ANT [147]. One of the main mechanisms by which ANT plays a protective role in DN is by inhibiting the inflammatory response induced by the LXRα pathway and blocking cholesterol deposition [148]. Flavonoids constitute a major class of polyphenolic compounds with diverse pharmacological activities. Flavonoids also have antifibrotic and antiapoptotic properties and play an important role in renoprotective effects in CKD by interfering with TGF-β1/Smad signaling and inhibiting the epithelial-to-mesenchymal transition [149]. Tannins, polyphenolic compounds from bilberries, play an important role in controlling the progression of diabetic microvascular complications. This will help researchers find ways to develop new cost-effective therapies for managing the complications of diabetes [14]. 

## 7. Clinical Evidence for the Effect of *Vaccinium* on Diabetes and Diabetic Microvascular Complications

Owing to the powerful antioxidant effects of *Vaccinium*, the therapeutic potential of these fruits and their extracts has been evaluated for several chronic diseases, including diabetes mellitus, cancer, and neurodegenerative and cardiovascular diseases. This review focuses on *Vaccinium* extracts for the treatment of diabetes and diabetic microvascular complications (DR and DKD).

### 7.1. Effect of Vaccinium on Type 2 Diabetes Mellitus Treatment

There are many clinical studies on the treatment of diabetes with *Vaccinium*, which can lower blood glucose levels. Clinical evidence for the anti-diabetic effects of *Vaccinium.* is listed in Table 4. Whole blueberry and soluble fiber supplementation prevents gestational weight gain, improves inflammation, and controls blood glucose levels in obese women [150]. In addition, in adults, pancreatic polypeptide (PP) concentrations were remarkably higher when 140 g of whole blueberries were administered [151]. The consumption of 22 g of freeze-dried blueberries for 8 weeks was beneficial to the hearts of men with T2D [152]. In addition to blueberries, bilberries, cranberries, and whortleberries have a similar effect on blood sugar control, and some studies have recommended the use of bilberries to regulate blood glucose levels in patients with T2D or metabolic syndrome [153,154,155]. In addition, one study showed that bilberries lower postprandial blood glucose and insulin levels [155]. One study showed that cranberries could improve postprandial glucose management [156]. In addition, dried cranberries [27] and cranberry juice [157,158,159] have similar effects and that whortleberry extract considerably decreases HbA1c, fasting glucose, and 2 h postprandial glucose levels [160]. ANT are chemicals found in *Vaccinium* species. Purified ANT favorably affects glycemic control and the lipid profile [161,162].

A recent meta-analysis showed that consumption of blueberries and cranberries remarkably reduced fasting blood glucose and glycated hemoglobin levels in patients with diabetes is highly credible. In individuals with diabetes, the consumption of cranberries or blueberries considerably reduced fasting blood glucose [MD: −17.72 mg/dL; 95% CI: −29.62, −5.82; *p* = 0.03; I2 = 57%] and glycated hemoglobin [MD: −0.32; 95% CI: −0.57, −0.07; *p* = 0.15; I2 = 39%]; however, there was no effect on insulin resistance [37]. Similarly, another meta-analysis, including seven randomized controlled trials, involved 270 adult patients with T2D, who consumed cranberry juice (240 mL) daily for 12 weeks and were supplemented with powder or blueberry extract (9.1–9.8 mg of ANT) for 8 to 12 weeks to control blood glucose in patients with T2D, despite the heterogeneity in the form of dose, administration (natural, extract, dried, preparation-juice), duration of intervention, and type of population studied involving these two berries [36]. Grohmann et al. [39] showed that interventions with lingonberry and blackcurrant extracts resulted in a mean reduction in HbA1c and fasting glucose levels of 4.7% and 3%, respectively, and that lingonberry and blackcurrant extracts were beneficial for glucose metabolism, although the current evidence is supported by only a few studies in Chinese subjects with T2DM.

In clinical trials using *Vaccinium* specifically, oral administration of the fruit and its extracts has shown mixed results. Owing to the high amount of sugar present in *Vaccinium*, extracts without sugar tend to show better anti-diabetic effects than the whole fruit or juice because of the higher content of bioactive substances [23]. A study in which patients consumed 400 g of fresh bilberries for eight weeks showed a negative correlation between the dietary intake of lingonberries and fasting plasma glucose levels; however, insulin sensitivity remained unchanged [163]. 

However, other clinical studies have shown no significant differences in fasting glucose levels between treatment and control groups after 12 [164] or 24 weeks [165] of dietary anthocyanin supplementation, or 2 months of daily intake of 400 g of fresh lingonberries [166]. Even in the latest clinical study in Chinese patients with T2DM, using 1.4 g of bilberry extract daily for 6 weeks, HbA1c decreased by 0.31 ± 0.58% while taking the supplement; however, this change was not considerably different compared to placebo, and there was also no considerable difference between lingonberry extract and placebo in antioxidant status, oxidative stress and inflammatory status treatment [28]. 

### 7.2. Research for the Treatment of DR and DKD

There are few clinical studies on *Vaccinium* and its extracts in the treatment of DR. In the first open-label placebo-controlled study of bilberry extract in DR, a combination of 200 mg bilberry extract and 10 mg carotene administered thrice a day reduced vascular permeability and improved retinal vascularity [167]. In another study, in patients with diabetic and hypertensive retinopathy, 160 mg of bilberry extract containing 25% ANT taken twice daily showed a 77–90% improvement in fundus examination and fluoroscopic angiographic abnormalities compared with placebo [168]. One study tested the effect of bilberry fruit extract on patients with diabetic retinopathy at a dose of 510 mg/day for one year, with gradual improvement in contrast sensitivity, but other measured parameters (corrected visual acuity, microaneurysms, hard exudates, and leaking points) remained unchanged for the entire duration of the study [169]. In a randomized, double-blind, monocentric, prospective study, supplementation with Macuprev (containing bilberries 36% and anthocyanosides 90 mg) increased the function of macular preganglional components, which helped to decrease inflammation in DR lesions [170]. Bilberries are also used to treat diabetes and microvascular complications [171]. However, high HDL levels are also associated with diabetic retinopathy [172]. Therefore, more basic experiments are needed to understand the mechanisms by which HDL affects DR. Further clinical trials are required.

Although there are few clinical studies on *Vaccinium* in the treatment of DKD, *Vaccinium* and its active components have shown promising results in the clinical intervention of CKD. *Vaccinium* is an important component of local diets in many countries. It is popular because of its pleasant taste and is often processed into alcoholic beverages, preservatives, jams, pies, and juices. Plant-based diets may help manage and prevent some of the symptoms and metabolic complications of CKD [173]. In a meta-analysis of cohort studies on CKD, seven studies including 15,285 participants showed that a plant-based diet reduced the risk of CKD [174]. There is growing evidence that an entire plant-based diet may slow the progression of CKD, reduce the incidence of cardiovascular disease, and lower the rates of obesity and diabetes, which, in turn, may delay the onset of kidney failure and dialysis [175,176,177].

In addition, some clinical studies have demonstrated the vascular protective effects of *Vaccinium* (Table 5), indicating its potential application in the prevention of diabetic microvascular complications. One study showed the first sustained improvements in lipid status, vascular function, and underlying NO bioactivity following consumption of one cup of blueberries per day [178]. These findings suggest that blueberries exert immunomodulatory effects and reduce oxidative stress and inflammation in patients with metabolic syndrome [179]. Among the *Vaccinium* species, blueberry, cranberry, and bilberry have this vascular protective function. Cranberries decrease atherosclerotic cholesterol profiles, including total and LDL-C levels, and the total-to-HDL cholesterol ratio [180]. These findings suggest that daily consumption of cranberry beverages for 8 weeks may help reduce lipid status and alter certain biomarkers of oxidative stress in individuals with obesity and a pro-inflammatory state [181].

Despite progress in studies on the hypolipidemic and hypoglycemic effects of *Vaccinium* and improvement in DR, further studies with larger cohorts, longer follow-up periods, and more reliable endpoints (for example, proteinuria, glomerular filtration rate, and disease progression) are required to evaluate the use of lingonberry extract as an add-on therapy for T2D, diabetic retinal disease, and glycogenic kidneys.

## 8. Conclusions and Future Perspectives

Diabetes mellitus and its microvascular complications require effective dietary supplement adjuvant therapy. Because *Vaccinium* fruit contains many antioxidant compounds, clinical application of *Vaccinium* extract as nutritional health products may be beneficial for diabetes-related microvascular complications, especially DKD and DR. Compared with common drugs, the *Vaccinium* extract is currently safe and mostly has no side effects. *Vaccinium* extracts offer a means to discover and develop new drugs; however, they have some drawbacks. *Vaccinium* does not deliver the intense and potent therapeutic effects of drugs such as SGLT2. *Vaccinium* is generally studied as a whole, the exact components that affect the disease are unknown, and the understanding of the underlying mechanism is still in its infancy. The limitation of clinical usefulness is poor bioavailability. In clinical trials, juice is available in 240 mL [159,160] or 480 mL [157] daily, bilberry supplements are available in doses of 1 g [153] or 1.4 g [28] daily, and the dosage of the whole fruit of blueberries is 140 to 300 g [150,151,152,178] daily. The best dose of *Vaccinium* is unknown; it is usually found in juice, fruits, extract, and other forms, and more experiments are needed to determine the dosage. Although ANT are rapidly absorbed, they are also rapidly metabolized and excreted from the body [183,184].

Current technological developments in the pharmaceutical industry are driving the development of *Vaccinium* extracts for T2D and its associated microvascular complications, allowing for increased purity percentages and optimized formulations to obtain greater in vivo stability and target tissue bioavailability, thereby prolonging their therapeutic effects. 

This makes *Vaccinium* a promising treatment for diabetes and diabetic microvascular complications. However, further studies on the mechanisms involved, as well as larger randomized blinded trials, are urgently required.

## Figures and Tables

**Table 1:** The main flavonoids in four popular *Vaccinium* species.

Species	Geographical Sources	Anthocyanins	Other Flavonoids
*V. myrtillus* (bilberry) [19,30]	Central and northern parts of Europe	cyanidin 3-galactoside, cyanidin 3-glucoside, cyanidin 3-arabinoside, delphinidin 3-galactoside, delphinidin 3-arabinoside, delphinidin 3-glucoside, malvidin 3-galactoside, malvidin 3-arabinoside, malvidin 3-glucoside, petunidin 3-galactoside, petunidin 3-arabinoside, petunidin 3-acetylglucoside, petunidin 3-glucoside, peonidin 3-galactoside, peonidin 3-arabinoside, cyanidin 3-xyloside, cyanidin 5-glucoside, cyanidin 3,5-diglucoside, cyanidin 3-(6″-O-2-rhamnopyranpsyl-2″-O-β-xylopranosyl-β-glucopyranoside), cyanidin 3-sambubioside, delphinidin 3-sambuobiside, peonidin-3-glycoside	myricetin 3-glucoside, myricetin 3-arabinoside, myricetin3-rhamnoside, quercetin 3-arabinoside, quercetin 3-rhamnoside, quercetin 3-galactoside, quercetin 3-glucoside, quercetin 3-rutinoside, apigenin, chrysoeriol, myricetin, myricetin-3-xyloside, quercetin 3-glucuronide, quercetin 3-xyloside, isorhamnetin 3-glucoside, isorhamnetin 3-xyloside isorhamnetin, laricitrin, syringetin, luteolin, kaempferol isorhamnetin 3-galactoside, myricetin 3-glucuronide, laricitrin 3-glucoside, syringetin 3-glucoside, kaempferol 3-glucoside, myricetin 3-galactoside,
*V. corymbosum* (blueberry) [19,32,33]	Parts of Asia and North America	delphinidin 3-galactoside, delphinidin 3-glucoside, cyanidin 3-galactoside, delphinidin 3-arabinoside, cyanidin 3-glucoside, petunidin 3-galactoside cyanidin 3-arabinoside, petunidin 3-glucoside, peonidin 3-galactoside, petunidin 3-arabinoside malvidin 3-galactoside, malvidin 3-glucoside peonidin 3-arabinoside, malvidin 3-arabinoside, delphinidin 3-acetylglucoside, petunidin 3-acetylglucoside, malvidin 3-acetylglucoside, petunidin 3-glucoside	myricetin 3-galactoside, myricetin 3-glucoside, myricetin 3-rhamnoside, quercetin 3-galactoside, quercetin 3-glucoside, quercetin 3-xylosylglucuronide, quercetin 3-glucosylxyloside, quercetin 3-rutinoside, quercetin 3-acetylrhamnoside, quercetin 3-xyloside
*V. vitis idaea* (lingonberry) [30,34]	North Eurasia and North America	cyanidin 3-glucoside, delphinidin 3-glucoside, cyanidin 3-arabinoside, peonidin 3-arabinoside, peonidin 3-glucoside, peonidin 3-galactoside, delphinidin 3-arabinoside, delphinidin 3-galactoside, petunidin 3-galactoside, petunidin 2-glucoside, malvidin 3-galactoside, malvidin 3-glucoside	kaempferol, quercetin, myricetin, rutin myricetin 3-glucoside, quercetin 3-glucoside, quercetin 3-galactoside, quercetin 3-arabinoside, quercetin 3-xyloside, kaempferol 3-rhamnoside, quercetin 3-rhamnoside, isorhamnetin 3-galactoside, isorhamnetin 3-glucoside, syringetin 3-glucoside, kaempferol 3-glucoside
*V. macrocarpon* (cranberry) [33,35]	Eastern US and Canada	cyanidin-3-glucoside, cyanidin-3-galactoside, cyanidin-3-arabinoside, peonidin-3-glucoside, peonidin-3-galactoside, peonidin-3-arabinoside, pelargonidin-3-galactoside, pelargonidin-3-arabinoside, malvidin-3-galactoside, malvidin-3-arabinoside delphinidin-3-arabinoside, petunidin-3-galactoside	kaempferol-3-glucoside, quercetin-3-galactoside, quercetin-3-arabinoside, quercetin-3-rhamnoside

**Table 2:** Experimental studies on the treatment of diabetes mellitus with *Vaccinium* extracts.

	Animal	Intervention	Duration	Results
Takács et al. 2020 [100]	Normal (control), obese, prediabetic, and streptozotocin-induced diabetic mice	Wild strawberry (Fragaria vesca), blackberry (Rubus fruticosus), and European blueberry (*Vaccinium* myrtillus) leaf extracts	/	Inhibit α-glucosidase and α-amylase enzyme activity in vitro, attenuated the starch-induced rise of blood glucose levels.
Mykkänen et al. 2014 [122]	C57BL mice fed with a high-fat diet (HFD)	The effects of 5% or 10% (*w*/*w*) of whole bilberries (BB)	3 months	Prevented ameliorated type 1 pro-inflammatory responsiveness, blood pressure
Feshani et al. 2011 [123]	Alloxan-diabetic male Wistar rats	Fruit of *Vaccinium* arctostaphylos L. (Ericaceae)	3 weeks	Decreased postprandial blood glucose and TG, increased erythrocyte superoxide dismutase, GPX, and catalase.
Prior et al. 2010 [112]	Male C57BL/6J mice (25 days of age) fed with HFD or LFD (low-fat diet)	Blueberry juice or purified blueberry ANT (0.2 or 1.0 mg/mL) in the drinking water	72 days	Blueberry juice was not as effective as the low dose of ANT in the drinking water in preventing obesity.
Zhong et al. 2020 [113]	HFD-fed mice	BBJ, and FBJ with homemade probiotic starter or CFBJ	17 weeks	All decreased fat accumulation and LDL-C levels. BBJ and FBJ treatments regulated the liver mRNA and protein expression levels involved in lipid and glucose metabolism.
Anhê et al. 2015 [118]	C57BL/6J mice were fed either a chow or a high fat/high sucrose diet	Daily either with vehicle (water) or cranberry extract (200 mg/kg)	8 weeks	Cranberry extract reduced weight gain and visceral obesity, improve insulin sensitivity, lowered intestinal TG content, increased the proportion of the mucin-degrading bacterium Akkermansia.
Brader et al. 2013 [124]	Male Zucker Diabetic Fatty (ZDF) rats	A control, bilberry-enriched, blackcurrant-enriched, or fiber-enriched diet	8 weeks	Bilberry enrichment ameliorated total and LDL but not HDL
Eid et al. 2014 [104]	C57BL/6 mice fed a HFD	Lingonberry extract to HFD at three different concentrations (125, 250, and 500 mg/Kg)	8 weeks	Decreased glycemia and strongly tended to decrease insulin levels, improved hepatic steatosis by decreasing hepatic TG levels and significantly activated liver AMPK and Akt pathways.
Al Hamimi et al. 2017 [105].	C57BL/6J fed a HFD	Control, two of which containing lingonberries (L1D and L2D) from different sources,	/	Glycemia was improved only in mice fed L1D, both L1D and L2D liver function was improved, and inflammation reduced. Increased phosphatidylcholines and lysophosphatidylcholines, decreased serine and sphingomyelins
Madduma Hewage et al. 2020 [125]	Mice (C57BL/6J) fed a HFD	Dietary supplementation of lingonberry	12 weeks	Decreased BUN, KIM-1, NGAL, NF-κB, MCP-1, TNF-α, IL-6.
Vendrame et al. 2015 [114]	The obese Zucker rat (OZR)	Fed an 8% enriched wild blueberry diet or a control (C) diet	8 weeks	Decreased plasma concentrations of glucose, insulin, glycated hemoglobin GHbA1c, resistin, and retinol-binding protein 4 (RBP4), compared to control diet.
Ryyti et al. 2021 [126]	C57BL/6N male mice	Fed with either a high-fat (HF) or low-fat (LF) diet or HF diet supplemented with air-dried lingonberry powder (HF + LGB).	6 weeks	Lingonberry supplementation prevented the effect of HF diet on an array of genes, such a Mogat1, Plin4, Igfbp2, Lcn2, Saa1, Saa2, Cxcl14, Gcp1, S100a10, Cdkn1a, Tubb2a, and Tubb6.
Khanal et al. 2010 [115]	Rats fed a high fructose diet	Dietary treatments were control (starch based), high fructose (HF), and HF containing either 3.3, 6.6, or 33 g cranberry powder/kg diet.	/	Fed with cranberry powder decreased plasma glucose and triglycerides, lower fasting plasma insulin.
Chen et al. 2020 [103]	ZDF rats	Fed with the nonacylated anthocyanin extract from bilberries (NAAB) or the acylated anthocyanin extract from purple potatoes (AAPP)	Daily doses of 25 and 50 mg/kg body weight for 8 weeks	NAAB reduced fasting plasma glucose level, the levels of branched-chain amino acids and improved lipid profiles.
Pemmari et al. 2022 [110]	HFD-induced mouse model of obesity	Air-dried bilberry powder	/	The bilberry supplementation was unable to modify the weight gain, prevented the increase in the hepatic injury marker ALT and many inflammatory factors like SAA, MCP1, and CXCL14, prevented the increase in serum cholesterol, glucose, and insulin levels.
Petersen et al. 2022 [116]	Seven-week-old diabetic db/db mice	Standard diet (db/db) or a diet supplemented with 3.8% freeze-dried blueberry (db/db + BB)	10 weeks	Blueberry supplementation reduces NOX4 and IκKβ, increases commensal microbes.
Takikawa et al. 2010 [127]	T2D mice	The effect of dietary bilberry extract (BBE)	/	Ameliorates hyperglycemia and insulin sensitivity via activation of AMP-activated protein kinase (AMPK).
Medina-Larqué et al. 2022 [119]	C57BL6 male mice fed an obesogenic high-fat and high-sucrose (HFHS) diet	Cranberry polyphenols (CP), agavins (AG), CP + AG	9 weeks	Both CP and AG can shape gut microbiota composition and regulate key mucosal markers involved in the repair of epithelial barrier integrity.
Zhou et al. 2020 [120]	Male C57BL/6J mice fed with normal diet or HFD	Cranberry polyphenolic extract	16 weeks	CPE reduced but did not normalize HFD-induced body weight gain.
Singh et al. 2018 [121]	Male Swiss albino mice were fed normal chow or HFD	Administered either cranberry extract (CRX) (200 mg/kg) alone or in combination with isomalto-oligosaccharides (IMOs) (1 g/kg)	/	Combination of CRX and IMOs prevented systemic and tissue inflammation, glucose intolerance, and systemic obesity-associated metabolic changes in adipose tissue and liver.
Nair et al. 2014 [117]	Five-week-old lean and obese Zucker rats (LZR and OZR)	Fed a blueberry-enriched diet or an isocaloric control diet	15 weeks	Blueberry (BB) protects by inhibiting TLR4.
Lee et al. 2018 [128]	Twenty-four male Wistar rats	Fed low-fat (LF; 10% fat), HF or HF with 10% by weight blueberry powder diets	8 weeks	Increase Gammaproteobacteria abundance. Ileal villus height, tumor necrosis factor α (Tnfa) and interleukin 1β (Il1b) gene expression normalized by blueberry supplementation. improved markers of insulin sensitivity.
Morissette et al. 2020 [129]	Sixty-eight C57BL/6 male mice	Balanced diet (Chow); high-fat, high-sucrose diet (HFHS); or HFHS supplemented with whole blueberry powder (BB), anthocyanidin (ANT)-rich extract, or proanthocyanidin (PAC)-rich extract	12 weeks	PAC-treated mice were leaner, improved insulin responses during OGTT.
Seymour et al. 2011 [130]	Zucker Fatty and Zucker Lean rats	Fed a HFD or LFD containing 2% (*wt*/*wt*) freeze-dried whole highbush blueberry powder	/	The addition of blueberry reduced triglycerides, fasting insulin, homeostasis model index of insulin resistance, and glucose area under the curve, reduced abdominal fat mass.
Nunes et al. 2021 [131]	Hypercaloric diet-induced prediabetic rat model	Blueberry juice (BJ)	14 weeks	Counteracted diet-evoked metabolic deregulation, improving glucose tolerance, insulin sensitivity, and hypertriglyceridemia, along with systemic and hepatic antioxidant properties, alleviated hepatic steatosis and mitochondrial dysfunction.
Elks et al. 2015 [132]	Four-week-old female C57BL/6J mice after induction of menopause	Fed a high-fat diet or the same diet supplemented with 4% blueberries (BB) powder	12 weeks	BB supplementation prevents the glucose intolerance and hepatic steatosis, and these effects are independent of body weight.
Wu et al. 2018 [133]	HFD fed C57BL/6 mice	LFD, HFD, or HFD plus orlistat, and blackberry anthocyanins (BLA) or blueberry anthocyanins (BBA) in their daily food	12 weeks	Reduced serum and hepatic lipid levels and increased hepatic superoxide dismutase and GPx, activities, attenuated expression of tumor necrosis factor TNF-α, interleukin-6, and nuclear factor-kappaB genes.
Ryyti et al. 2020 [134]	Thirty male C57BL/6N mice	Receive LF, HF and lingonberry-supplemented high-fat (HF + LGB) diet	6 weeks	Lingonberry supplementation prevented adverse changes blood cholesterol and glucose levels, restrained proinflammatory adipocytokine leptin, increase serum amyloid A.
Vuong et al. 2009 [135]	KKA(y) mice	Biotransformed blueberry juice (BJ)	/	BJ decreases hyperglycemia, in part by reversing adiponectin levels, protects young pre-diabetic mice from developing obesity and diabetes.
Wu et al. 2016 [136]	HFD induced obese male C57BL/6 mice	Blueberry anthocyanin (BA) at doses of 50, 100, and 200 mg/kg	8 weeks	BA at high doses reduced body weight, low and middle doses did not affect. A high dose could effectively decrease serum glucose, attenuate epididymal adipocytes, improve lipid profiles, and significantly down-regulate expression levels of TNFα, IL-6 PPARγ, and FAS genes.

**Table 3:** Experimental studies on DR and DKD treatment with *Vaccinium* extracts.

	Animal/Cell	Intervention	Duration	Results
Wang et al. 2015 [137]	Visible light-induced damage in human retinal pigment epithelial (RPE) cells	Four ACNs, pelargonidin-3-glucoside (Pg-3-glu), cyanidin-3-glucoside (Cy-3-glu), delphinidin-3-glucoside, and malvidin-3-glucoside (Mv-3-glu) from blueberry, blackberry and strawberry	/	Cy-3-glu exhibited the highest reactive oxygen species inhibitory capacity, Cy-3-glu and Mv-3-glu decrease VEGF, Cy-3-glu and Pg-3-glu inhibited the increase in β-galactosidase.
Li et al. 2022 [138]	HG-treated ARPE-19 cells	10 μM Cy-3-glu (blueberry extracts from northeast China)	/	Cy-3-glu ameliorating oxidative stress-induced BRB damage via the Nrf2 pathways.
Wang et al. 2022 [139].	Human retinal pigment epithelium cell line ARPE-19 cells were exposed to high concentration glucose (H-Glu) with 25 mM for 24 h	Blueberry anthocyanin extract (BAE)		The increase of apoptosis, ROS level and ERS in ARPE-19 cells induced by H-Glu was notably restored by BAE.
Song et al. 2016 [140]	Intraperitoneal injection of streptozotocin (STZ, 60 mg/kg) was used to induce a rat diabetes model.	Blueberry anthocyanins at 20, 40, and 80 mg/kg were given orally	12 weeks	Blueberry anthocyanins prevent diabetes-induced weight loss and increased blood glucose, increased GSH and GPx, decreased MDA, ROS, VEGF and IL-1β, increased the mRNA levels of Nrf2 and HO-1.
Huang et al. 2018 [141]	high glucose- (HG-) induced injury in human retinal capillary endothelial cells (HRCECs)	Blueberry anthocyanin extract and its predominant constituents, malvidin (Mv), malvidin-3-glucoside (Mv-3-glc), and malvidin-3-galactoside (Mv-3-gal),	24 h	All increased cell viability, SOD he enzyme activity of catalase; decreased ROS.
Kim et al. 2015 [143]	streptozotocin-induced diabetic rats	Bilberries extract (100 mg/kg)	6 weeks	Bilberries extract did not affect the blood glucose levels and body weight; reduced the fluorescein leakage; decreased markers of diabetic retinopathy, such as retinal VEGF expression and degradation of zonula occludens-1, occludin, and claudin-5.
Stevens et al. 2019 [144]	a model of type II DN. Diabetic db/db mice	Administered DIAVIT in their drinking water	14 weeks	DIAVIT prevented albuminuria and glomerular water permeability; alters VEGF-A splicing in type II DN, rescuing the DN phenotype.
Di Cerbo et al. 2018 [145]	34 client-owned, neutered cats with II-III CKD	Control diet (*n* = 17) or a nutraceutical diet (*n* = 17, contain 0.0371% cranberry)	90 days	creatinine, blood urea nitrogen, total proteins, aspartate aminotransferase, urine turbidity score, color score, and total proteins decreased in cats that received the ND.
Li et al. 2022 [146]	6-week-old male C57BLKS/J-Lepr^db^/Lepr^db^ mice	10 mg/kg Cyanidin-3-*O*-glucoside per day by oral gavage	12 weeks	The fasting blood glucose level, perimeter of glomerular lesions, perimeter of glomerular lesions and kidney function (Cystatin C, urine creatinine) alleviated after ANT treatment compared to untreated; upregulated taurine, hypotaurine metabolism pathway tryptophan metabolism and tyrosine metabolism.
Qin et al. 2018 [147]	DN in db/db mice	Cyanidin 3-glucoside	/	Cyanidin 3-glucoside reduced body weight, the levels of blood urea nitrogen (BUN), serum creatinine, urinary albumin content and albumin/creatinine ratio (ACR);reduced the surface area of Bowman’s capsule, glomerular tuft, Bowman’s space, and decreased renal expression of collagen IV, fibronectin, transforming growth factor β 1 (TGFβ1), matrix metalloprotein 9 (MMP9) and α-smooth muscle actin (α-SMA), the Lee’s index, perirenal white adipose tissue weight, and high levels of blood and renal triglyceride and cholesterol, reduced systemic levels and renal expression of TNFɑ, IL-1ɑ, and monocyte chemotactic protein-1 (MCP-1); increased GSH; decreased GSSG level.
Du et al. 2015 [148]	High-glucose (HG)-stimulated HK-2 cells	ANTs:cyanidin-3-O-β-glucoside chloride [C3G] or cyanidin chloride [Cy]		Enhanced cholesterol efflux and ABCA1 expression; increased peroxisome proliferator-activated receptor alpha (PPARα) and liver X receptor alpha (LXRα) expression and decreased the HG-induced expression of the proinflammatory cytokines intercellular adhesion molecule-1 (ICAM1), monocyte chemoattractant protein-1 (MCP1), and transforming growth factor-β1 (TGFβ1), as well as NFκB activation, blocking cholesterol deposition and inhibiting the LXRα pathway-induced inflammatory response.

**Table 4:** Clinical evidence on anti-diabetic effects of *Vaccinium*.

	Sample	Age (y)	Intervention	Duration	Results
Basu et al. 2021 [150]	34 women at high risk of developing GDM	27 ± 5	280 g whole blueberries and 12 g soluble fiber daily or standard prenatal care	18 weeks	Lower maternal weight gain, C-reactive protein, and blood glucose based on GCT in intervention group, compared to the control group.
Mirfeizi et al. 2016 [153]	105 T2DM patients	30–65	Bilberry supplements 1 g or placebo daily	90 days	Reduced FBG, 2 h blood postprandial glucose and homeostasis model assessment of insulin resistance (HOMA-IR) scores in the bilberry group, compared with placebo group.
De Mello et al. 2017 [154]	47 individuals with metabolic syndrome	25–60	200 g of bilberry purée and 40 g of dried bilberries (altogether eq. 400 g of fresh bilberries) or control	8 weeks	Significant increase in fasting serum hippuric acid in intervention group, compared to the control group.
Wilson et al. 2010 [27]	13 noninsulin- dependent diabetics	61.6 ± 2.3	Sweetened dried cranberries (40 g, 113 cal, 1.8 g fiber, 10 g polydextrose)	/	Favorable glycemic and insulinemic response in intervention group.
Chan. et al. 2021 [28]	20 T2DM patients	55.8 ± 9.5	Bilberry supplementation (1.4 g of extract) or placebo daily	4 weeks	Tendency of improved glycemic control in intervention group, compared to the placebo group.
Stote et al. 2019 [151]	17 healthy adults	22–65	140 g of whole blueberries or placebo daily	/	Significant increase in pancreatic polypeptide (PP) concentrations in intervention group, compared to the placebo group.
Schell et al. 2017 [156]	25 T2DM patients	56 ± 6	Fast-food style high-fat breakfast (70 g fat, 974 kcal) with or without cranberries (40 g).	/	Lower postprandial increases of glucose at 2 and 4 h in the cranberry group, compared to control group.
Kianbakht. et al. 2013 [160]	37 T2DM patients	40–60	1050 mg of Caucasian whortleberry fruit hydroalcoholic extract or placebo daily, in combination with anti-hyperglycemic drugs	2 months	Lower blood levels of fasting glucose, 2 h postprandial glucose, and HbA1c in intervention group, compared to the placebo group.
Novotny et al. 2015 [157]	56 individuals	25–65	480 mL of low-calorie cranberry juice or placebo daily	8 weeks	Reduced circulating TGs, CRP, and glucose, insulin resistance, and diastolic BP in intervention group, compared to the placebo group.
Hoggard et al. 2013 [155]	8 male volunteers with T2D	62 ± 5	A single capsule of 0.47 g standardized bilberry extract (36% (*w*/*w*) anthocyanins) (eq. 50 g of fresh bilberries) or placebo followed by a polysaccharide drink (eq. 75 g glucose)	/	Reduced postprandial glycaemia and insulin levels in intervention group, compared to the placebo group.
Wilson et al. 2008 [158]	12 T2DM patients	65.3 ± 2.3	Unsweetened low-calorie cranberry juice (LCCBJ; 19 Cal/240 mL) and control	/	Favorable metabolic response in intervention group, compared to the control group.
Shidfar et al. 2012 [159]	58 male volunteers with T2D	54.8 ± 9.1	240 mL of cranberry juice or placebo daily	12 weeks	Significant decrease in serum glucose and apo B; and significant increase in serum apoA-1 and PON-1 activity in intervention group, compared to the placebo group.
Yang et al. 2017 [161]	160 participants with prediabetes or early untreated diabetes	40–75	Purified anthocyanins (320 mg/day) or placebo	12 weeks	Reduced HbA1c, low-density lipoprotein-c, apolipoprotein A-1, apolipoprotein B in intervention group, compared to the placebo group.
Li et al. 2015 [162]	58 diabetic patients	56–67	160 mg of anthocyanins twice daily or placebo	24 weeks	Decreased serum LDL cholesterol, triglycerides, apolipoprotein B, and apo C-III; increased HDL cholesterol; higher total radical-trapping antioxidant parameter and ferric ion reducing antioxidant power values; lower fasting plasma glucose, homeostasis model assessment for insulin resistance index, and elevated serum adiponectin and b-hydroxybutyrate in intervention group, compared to the placebo group.
Stote et al. 2020 [152]	58 male volunteers with T2D	51–75	22 g freeze-dried blueberries or placebo daily	8 weeks	Lower hemoglobin A1c, fructosamine, triglycerides, aspartate transaminase, and alanine transaminase levels in intervention group, compared to the placebo group.

**Table 5:** Clinical evidence on vascular protective effects of *Vaccinium*.

	Sample	Age (y)	Intervention	Duration	Results
Curtis et al. 2019 [178]	115 adults with MetS	63 ± 7	Blueberries (75 g or 150 g) or placebo daily	6 months	Improvements in vascular function, lipid status, and underlying NO bioactivity in intervention group, compared to the placebo group.
Nair et al. 2017 [179]	27 adults with metabolic syndrome	/	Blueberries (45 g freeze-dried) or placebo daily	6 weeks	Decreased superoxide and total ROS in whole blood and monocytes; increased myeloid DC; decreased monocyte gene expression of TNFα, IL-6, TLR4 and reduced serum GMCSF in intervention group, compared to the placebo group.
Hsia et al. 2020 [181]	35 individuals with obesity and with elevated fasting glucose or impaired glucose tolerance	/	450 mL of low-energy cranberry beverage or placebo daily	8 weeks	Levels of 8-isoprostane (biomarker of lipid peroxidation) decreased in the cranberry group but increased in the placebo group.
Lehtonen et al. 2011 [182]	110 female overweight and obese women	44.2 ± 6.2	Bilberry diets (equivalent to an average daily dose of 100 g fresh bilberries)	33–35 days	Decrease in waist circumference, weight, and Vascular cell adhesion molecule (VCAM).
Lee et al. 2008 [180]	30 T2D subjects taking oral glucose-lowering drugs	65 ± 1	Three capsules (500 mg/capsule) of cranberry extracts or placebo daily	12 weeks	Reduced atherosclerotic cholesterol profiles, including LDL-C, total cholesterol, and total: HDL cholesterol ratio in intervention group, compared to placebo group.

## Data Availability

Not applicable.

## Abbreviations

ACEI, Angiotensin-converting enzyme inhibitors; AGEs, advanced glycation end products; AMPK, adenosine monophosphate-activated protein kinase; Ang-2, Angiopoietin-2; ANT, Anthocyanins; aP2, adipocyte protein 2; ARB, Angiotensin Receptor Blocker; BA, blueberry anthocyanin; BAE, blueberry anthocyanin extract; BBE, bilberry extract; BBJ, blueberry juice; BRB, blood retinal barrier; CANVAS, Canagliflozin Cardiovascular Assessment Study; CFBJ, commercial fermented blueberry juice; CKD, chronic kidney disease; CoA, coenzyme A; CTGF, connective tissue growth factor; CVD, cardiovascular disease; DGAT1, diacylglycerol acyltransferase-1; DIO, diet-induced obesity; DKD, diabetic kidney disease; DM, diabetes mellitus; DN, diabetic nephropathy; DR, diabetic retinopathy; EMPA-REG Outcomes, Empagliflozin, Cardiovascular Outcomes, and Mortality in Type 2 Diabetes; eNOS, endothelial nitric oxide synthase; ERS, endoplasmic reticulum stress; ESRD, end-stage renal disease; FAS, fatty acid synthase; FBJ, fermented blueberry juice; FFA, free fatty acid; FGF-2, fibroblast growth factor-2; GBM, glomerular basement membrane; GFR, glomerular filtration rate; GPx, glutathione peroxidase; GSH, glutathione; HbA1, Hemoglobin alpha 1; HDL, High density liptein; HFD, high-fat diet; HO-1, heme oxygenase 1; LCCBJ, low-calorie cranberry juice; LDL, low density lipoprotein; LDL-C, low density lipoprotein cholesterol; LF, low-fat diet; IL-1, interleukin-1; IL-1β, interleukin-1β; IL-6, interleukin-6; IL-8, interleukin-8; LXRα, Liver X Receptor Alpha; IR, Insulin receptor; IRS, Insulin receptor substrate; MDA, malondialdehyde; MS, metabolic syndrome; NAAB, Nonacylated anthocyanins extract from bilberries; NLRP3, NOD-like receptor thermal protein domain associated protein 3; NO, nitric oxide; Nox, NADPH oxidase complex; NOX4, NADPH oxidase 4; NPDR, non-proliferative diabetic retinopathy; Nrf2, nuclear factor erythroid 2-related factor 2; OGG1, 8-oxoguanine-DNA glycosylase; OZR, obese Zucker rat; PDGF, Platelet-derived growth factor; PDR, proliferative diabetic retinopathy; PKC, protein kinase C; PP, pancreatic polypeptide; RAAS, Renin-angiotensin-aldosterone system; RNS, reactive nitrogen species; ROS, reactive oxygen species; SGLT2, sodium–glucose cotransporter 2; T2D, type 2 diabetes; T1D, type 1 diabetes; TG, triglycerides; TGF-β1, transforming growth factor beta 1; TLR2, Toll-like receptor 2; TLR4, Toll-like receptor 4; TNFα, tumor necrosis factor alpha; VEGF, vascular endothelial growth factor; VEGF-A, Vascular endothelial growth factor A; WoSCC, Web of Science Core Collection; ZDF, Zucker Diabetic Fatty.
